# Supplementary material for: Eleven genomic loci affect plasma levels of chronic inflammation marker soluble urokinase-type plasminogen activator receptor
Source: Commun Biol. 2021 Jun 2;4:655. doi: 10.1038/s42003-021-02144-8 (PMC8172928; doi:10.1038/s42003-021-02144-8)
Supplement: Supplementary file 2 — Description of Additional Supplementary Files [file 42003_2021_2144_MOESM2_ESM.pdf]

Description of Additional Supplementary Files

File name: Supplementary Data 1-15

Description:

Supplementary Data 1: Full Summary Statistics Table of the 13 independently genome-wide significant sequence variants associated with suPAR.

Supplementary Data 2: Comparison of Genetic Variants' Effects for suPAR Unadjusted For Smoking Vs Adjusted For Smoking.

Supplementary Data 3: Detailed Results Table from the Validation cohorts.

Supplementary Data 4a: suPAR associated variant rs2302524 in PLAUR variant information.

Supplementary Data 4b: suPAR associated variant rs2302524 Meta-analysis Combined summary statistics.

Supplementary Data 4c: suPAR associated variant rs2302524 Association Results in each separate cohort.

Supplementary Data 4d: suPAR associated variant rs2302524 Association Results in the Dunedin Validation cohort.

Supplementary Data 4e: suPAR associated variant rs2302524 Association Results in the E-Risk Validation cohort.

Supplementary Data 5: Results of the BiNGO biological network gene ontology overrepresentation analysis for biological processes, using the 12 genes found from the suPAR GWAS Meta-analysis.

Supplementary Data 6: Results from the suPAR PRS-based pheWAS with case control phenotypes in the Icelandic Population.

Supplementary Data 7: Results from the suPAR PRS-based pheWAS with quantitative phenotypes in the Icelandic Population.

Supplementary Data 8: Results from the suPAR PRS-based pheWAS with quantitative phenotypes in the UK Biobank.

Supplementary Data 9: Results from the Single-variant pheWAS with case/control phenotypes in the Icelandic population.

Supplementary Data 10: Results from the Single-variant pheWAS with case/control phenotypes in the UK Biobank.

Supplementary Data 11: Results from the Single-variant pheWAS with quantitative phenotypes in the Icelandic Population.

Supplementary Data 12: Results from the Single-variant pheWAS with quantitative phenotypes in the UK Biobank.

Supplementary Data 13: Results from Mendelian Randomization analyses for suPAR vs. the identified pheWAS Findings.

Supplementary Data 14: Cis-expression quantitative trait loci (eQTL) Analysis of the suPAR-associated variants.

Supplementary Data 15: Sources for expression quantitative trait loci (eQTL) data.
