# Supplementary material for: Eleven genomic loci affect plasma levels of chronic inflammation marker soluble urokinase-type plasminogen activator receptor
Source: Commun Biol. 2021 Jun 2;4:655. doi: 10.1038/s42003-021-02144-8 (PMC8172928; doi:10.1038/s42003-021-02144-8)
Supplement: Supplementary file 4 — Reporting Summary [file 42003_2021_2144_MOESM4_ESM.pdf]

nature research | reporting summary

nature research

Corresponding author(s): Joseph Dowsett
Last updated by author(s): Apr 20, 2021

Reporting Summary

Nature Research wishes to improve the reproducibility of the work that we publish. This form provides structure for consistency and transparency in reporting. For further information on Nature Research policies, see our Editorial Policies and the Editorial Policy Checklist.

Statistics

For all statistical analyses, confirm that the following items are present in the figure legend, table legend, main text, or Methods section.

n/a	Confirmed	
☐	☒	The exact sample size (*n*) for each experimental group/condition, given as a discrete number and unit of measurement
☒	☐	A statement on whether measurements were taken from distinct samples or whether the same sample was measured repeatedly
☐	☒	The statistical test(s) used AND whether they are one- or two-sided<br>*Only common tests should be described solely by name; describe more complex techniques in the Methods section.*
☐	☒	A description of all covariates tested
☐	☒	A description of any assumptions or corrections, such as tests of normality and adjustment for multiple comparisons
☐	☒	A full description of the statistical parameters including central tendency (e.g. means) or other basic estimates (e.g. regression coefficient) AND variation (e.g. standard deviation) or associated estimates of uncertainty (e.g. confidence intervals)
☐	☒	For null hypothesis testing, the test statistic (e.g. *F*, *t*, *r*) with confidence intervals, effect sizes, degrees of freedom and *P* value noted<br>*Give P values as exact values whenever suitable.*
☒	☐	For Bayesian analysis, information on the choice of priors and Markov chain Monte Carlo settings
☒	☐	For hierarchical and complex designs, identification of the appropriate level for tests and full reporting of outcomes
☐	☒	Estimates of effect sizes (e.g. Cohen's *d*, Pearson's *r*), indicating how they were calculated

Our web collection on statistics for biologists contains articles on many of the points above.

Software and code

Policy information about availability of computer code

Data collection	Variants in the Icelandic and Danish cohorts were imputed using software developed at deCODE genetics based on the IMPUTE HMM model [PMID: 17572673] as previously described [PMID: 25977816]. A linear mixed model implemented by BOLT-LMM [PMID:25642633] was used to test for association between sequence variants and suPAR levels.<br>We used publicly available software (URLs listed below) in conjunction with the above described algorithms in the sequencing processing pipeline (Whole-genome sequencing, Association testing, RNA-seq mapping and analysis):<br>BWA 0.7.10 mem, https://github.com/lh3/bwa<br>GenomeAnalysisTKLite 2.3.9, https://github.com/broadgsa/gatk/<br>Picard tools 1.117, https://broadinstitute.github.io/picard/<br>SAMtools 1.3, http://samtools.github.io/<br>Bedtools v2.25.0-76-g5e7c696z, https://github.com/arq5x/bedtools2/<br>Variant Effect Predictor https://github.com/Ensembl/ensembl-vep<br>BOLT-LMM https://data.broadinstitute.org/alkesgroup/BOLT-LMM/downloads/"<br>IMPUTE2 v2.3.1 https://mathgen.stats.ox.ac.uk/impute/impute_v2.html<br>dbSNP v140; http://www.ncbi.nlm.nih.gov/SNP/<br>LD Score Regression software; https://github.com/bulik/ldsc<br>BiNGO v3.0.3 https://www.psb.ugent.be/cbd/papers/BiNGO/Download.html<br>Cytoscape v3.7.1 https://cytoscape.org/download.html<br>We used R extensively to analyze data and create plots.
Data analysis	Variants in the Icelandic and Danish cohorts were imputed using software developed at deCODE genetics based on the IMPUTE HMM model [PMID: 17572673] as previously described [PMID: 25977816]. A linear mixed model implemented by BOLT-LMM [PMID:25642633] was used

to test for association between sequence variants and suPAR levels.
We used publicly available software (URLs listed below) in conjunction with the above described algorithms in the sequencing processing pipeline (Whole-genome sequencing, Association testing, RNA-seq mapping and analysis):
BWA 0.7.10 mem, https://github.com/lh3/bwa
GenomeAnalysisTKLite 2.3.9, https://github.com/broadgsa/gatk/
Picard tools 1.117, https://broadinstitute.github.io/picard/
SAMtools 1.3, http://samtools.github.io/
Bedtools v2.25.0-76-g5e7c696z, https://github.com/arq5x/bedtools2/
Variant Effect Predictor https://github.com/Ensembl/ensembl-vep
BOLT-LMM https://data.broadinstitute.org/alkesgroup/BOLT-LMM/downloads/"
IMPUTE2 v2.3.1 https://mathgen.stats.ox.ac.uk/impute/impute_v2.html
dbSNP v140; http://www.ncbi.nlm.nih.gov/SNP/
LD Score Regression software; https://github.com/bulik/ldsc
BiNGO v3.0.3 https://www.psb.ugent.be/cbd/papers/BiNGO/Download.html
Cytoscape v3.7.1 https://cytoscape.org/download.html
We used R extensively to analyze data and create plots.

For manuscripts utilizing custom algorithms or software that are central to the research but not yet described in published literature, software must be made available to editors and reviewers. We strongly encourage code deposition in a community repository (e.g. GitHub). See the Nature Research guidelines for submitting code & software for further information.

Data

Policy information about availability of data

All manuscripts must include a data availability statement. This statement should provide the following information, where applicable:
- Accession codes, unique identifiers, or web links for publicly available datasets
- A list of figures that have associated raw data
- A description of any restrictions on data availability

The suPAR meta-analysis summary statistics will be made available at https://www.decode.com/summarydata/.
Sequence variants passing GATK filters have been deposited in the European Variation Archive, accession number PRJEB15197.
For information on further access to data included in the meta-analysis, please contact the following authors of the respective cohorts: Hreinn Stefansson for data from the Icelandic cohort (hreinn.stefansson@decode.is) and Sisse Rye Ostrowski for data from the Danish Blood Donor Study (Sisse.Rye.Ostrowski@regionh.dk). The Dunedin Study data and E-Risk study data are not publicly available as approval for public data-sharing were not obtained from participants. The data are available on request by qualified scientists. Requests require a concept paper describing the purpose of data access, ethical approval at the applicant's institution, and provision for secure data access. Secure access is possible on the Duke University, Otago University, and King's College London campuses. For UK Biobank please register on https://bbams.ndph.ox.ac.uk/ams/ and apply for the data through there.

Field-specific reporting

Please select the one below that is the best fit for your research. If you are not sure, read the appropriate sections before making your selection.

- [x] Life sciences
- [ ] Behavioural & social sciences
- [ ] Ecological, evolutionary & environmental sciences

For a reference copy of the document with all sections, see nature.com/documents/nr-reporting-summary-flat.pdf

Life sciences study design

All studies must disclose on these points even when the disclosure is negative.

Sample size	The sample sizes for each cohort correspond to all available data from Iceland and Denmark (Danish Blood Donor Study) where genotype data and suPAR plasma levels were available, after quality control as specified in the Methods section.
Data exclusions	With the exception of standard, pre-established data quality control procedures specified in the Methods section, no data was excluded.
Replication	The variants were tested for heterogeneity between the Danish and Icelandic cohorts, of which the results showed remarkable consistency between the two cohorts. Only one variant, the rs71311394 intron variant in ST3GAL6, shows evidence of heterogeneity at P<0.05. However, direction of effects for rs71311394 are consistent between the two cohorts and the association with suPAR levels is significant in each cohort. Moreover, two independent cohorts agreed to validate the findings from this study: the Environmental Risk Longitudinal (E-Risk) Twin Study from Great Britain, as well as The Dunedin Longitudinal Study from New Zealand. Due to the replication cohorts' smaller N, some variants were not available for replication (especially for variants with very low allele frequencies or because of variants not surviving quality control). In the Dunedin cohort, five out of the eight available variants were confirmed and all eight variants had effect estimates in the same direction. In the E-Risk cohort, three out of the six available variants were confirmed and five variants had effect estimates in the same direction, although those that could not be confirmed in E-Risk may also be due to the fact that suPAR levels were measured at a young age (age 18).
Randomization	No randomization was used.

Blinding	Not relevant for this study, as this is a GWAS meta-analysis study.

Reporting for specific materials, systems and methods

We require information from authors about some types of materials, experimental systems and methods used in many studies. Here, indicate whether each material, system or method listed is relevant to your study. If you are not sure if a list item applies to your research, read the appropriate section before selecting a response.

Materials & experimental systems

n/a	Involved in the study
☒	☐ Antibodies
☒	☐ Eukaryotic cell lines
☒	☐ Palaeontology and archaeology
☒	☐ Animals and other organisms
☐	☒ Human research participants
☒	☐ Clinical data
☒	☐ Dual use research of concern

Methods

n/a	Involved in the study
☒	☐ ChIP-seq
☒	☐ Flow cytometry
☒	☐ MRI-based neuroimaging

Human research participants

Policy information about studies involving human research participants

Population characteristics

The following can also be found under Participants in the Methods Section.

Danish cohort: The Danish cohort is based on participants originating from the Danish Blood Donor Study (DBDS), a nationwide research platform utilizing the existing infrastructure in the Danish blood banks. Participants must be generally healthy and not on medication to be eligible as donors. Upon enrolment, participants gave informed consent, whole blood, plasma, and answered a comprehensive questionnaire. So far, approximately 110,000 adult DBDS participants have been enrolled with informed consent, whole blood, plasma samples, questionnaire data, and genome-wide genotype data gathered from each. suPAR was measured in 14,367 consecutive DBDS participants from March 1st 2010 until December 10th 2010 of which 12,177 (84.8%) participated in the GWAS after fulfilling quality control requirements.

Icelandic cohort: Plasma samples from 40,004 Icelanders were collected during 2000-2019. Fifty-two percent of the samples were collected as part of the Icelandic Cancer Project (ICP), while the remaining samples (48%) were collected as part of various genetic programs at deCODE genetics, Reykjavík, Iceland. In the ICP, all prevalent and newly diagnosed Icelandic cancer cases and their relatives were invited to participate in a comprehensive study of cancer, along with a control population, randomly selected from the National Registry. The median collection date for samples collected in conjunction with ICP was July 1st 2002, whereas the median collection date for other samples was May 15th 2015. All samples were measured using the SOMAscan platform (SomaLogic), containing 5,284 aptamers providing measurement of relative binding of the plasma sample to each of the aptamers in relative fluorescence units (RFU), corresponding to 4,792 proteins, of which suPAR is included. After quality control, unique measurements for N=35,559 individuals (88.9%) were used for genome-wide association analysis.

Environmental Risk (E-Risk) Longitudinal Twin Study: suPAR levels were measured at age 18 years in the E-Risk Study. Participants were members of the E-Risk Longitudinal Twin Study, which tracks the development of a 1994-95 birth cohort of 2,232 British children. Briefly, the E-Risk sample was constructed in 1999-2000, when 1,116 families (93% of those eligible) with same-sex 5-year-old twins participated in home-visit assessments. This sample comprised 56% monozygotic (MZ) and 44% dizygotic (DZ) twin pairs; sex was evenly distributed within zygosity (49% male). The sample represents socioeconomic conditions in Great Britain, as reflected in the families' distribution on a neighborhood-level socioeconomic index (ACORN [A Classification of Residential Neighborhoods], developed by CACI Inc. for commercial use): 25.6% of E-Risk families live in "wealthy achiever" neighborhoods compared to 25.3% nationwide; 5.3% vs. 11.6% live in "urban prosperity" neighborhoods; 29.6% vs. 26.9% in "comfortably off" neighborhoods; 13.4% vs. 13.9% in "moderate means" neighborhoods; and 26.1% vs. 20.7% in "hard-pressed" neighborhoods. (E-Risk underrepresents "urban prosperity" neighborhoods because such households are often childless). Home visits were conducted when participants were aged 5, 7, 10, 12, and most recently, 18 years (93% participation). At age 18, each twin was interviewed by a different interviewer. Whole blood was collected from 82% (n=1,700) of the participants. Plasma was available for 1,448 participants.

The Dunedin Multidisciplinary Health and Development Study: suPAR levels were measured at age 38 years in the Dunedin Study. Participants were members of the Dunedin Study, a longitudinal investigation of health and behavior in a representative birth cohort. Participants (n=1037; 91% of eligible births; 52% male) were all individuals born between April 1972 and March 1973 in Dunedin, New Zealand (NZ), who were eligible based on residence in the province and who participated in the first assessment at age 3 years. The cohort represented the full range of socioeconomic status (SES) in the general population of NZ's South Island and as adults matched the NZ National Health and Nutrition Survey on key adult health indicators (e.g., body mass index, smoking, GP visits) and the NZ Census of citizens of the same age on educational attainment. The cohort is primarily white (93%), matching South Island demographics. Assessments were carried out at birth and ages 3, 5, 7, 9, 11, 13, 15, 18, 21, 26, 32, and 38 years. At age 38 years, 95% (n=961) of the 1,007 participants still alive took part. At each assessment, each participant was brought to the research unit for interviews and examinations. Blood from participants of Maori ancestry was not transported to Duke University for cultural reasons, and plasma samples were not available for participants who did not provide blood or due to phlebotomy or defrost cycle problems.

Recruitment	See above.
Ethics oversight	Danish cohort:The project is approved by the Research Ethics Committees by the following 3 protocols: The Danish Blood Donor Study (M-20090237), Genetics of healthy ageing (CVK-1700407), Family study on the genetics of healthy ageing (NVK-1803847). The project is approved by the Danish Data Protection Agency under the combined approval for health care research at The Capital Region of Denmark (P-2019-99).<br>Icelandic cohort: All participants who donated samples gave informed consent and the National Bioethics Committee of Iceland approved the study (VSN-14-015) which was conducted in agreement with conditions issued by the Data Protection Authority of Iceland. Personal identities of the participants' data and biological samples were encrypted by a third-party system (Identity Protection System), approved and monitored by the Data Protection Authority.<br><br>E-Risk: The Joint South London and Maudsley and the Institute of Psychiatry Research Ethics Committee approved each phase of the study. Parents gave informed consent and twins gave assent between 5-12 years and then informed consent at age 18.<br>Dunedin: The relevant ethics committees approved each phase of the Study and written informed consent was obtained from all participants. The Otago University Ethics Committee, Duke University, and King's College London provided ethical approval for the Dunedin Study.

Note that full information on the approval of the study protocol must also be provided in the manuscript.
